# Oral Squamous Cell Carcinoma Clinico-pathological features in relation to Tumor Stage; AJCC 2018 perspective

**DOI:** 10.12669/pjms.39.2.7266

**Published:** 2023

**Authors:** Nauman Rauf Khan, Nadia Naseem, Nabeela Riaz, Rabia Anjum, Sobia Khalid, Asifa Iqbal, Saima Chaudhry

**Affiliations:** 1 Dr. Nauman Rauf Khan, BDS, M.Phil. Ph.D Scholar Dept. of Oral Pathology Department, University of Health Sciences, Lahore, Pakistan; 2 Dr. Nadia Naseem, MBBS, Ph.D., Dept. of Morbid Anatomy & Histopathology, University of Health Sciences, Lahore, Pakistan; 3 Dr. Nabeela Riaz, BDS, FCPS, FCPS., Chairperson, Department of Oral & Maxillofacial Surgery, King Edward Medical University, Mayo Hospital, Lahore, Pakistan; 4 Dr. Rabia Anjum, BDS, M.Phil., Assistant Professor, Department of Oral Pathology, University of Health Sciences, Lahore, Pakistan; 5 Dr. Sobia Khalid, MBBS, M.Phil., Consultant Pathologist, Siddique Family Hospital, Gujranwala, Pakistan; 6 Dr. Asifa Iqbal, BDS, M.Phil., Assistant Professor, Department of Oral Pathology, Rashid Latif Medical & Dental College, Lahore, Pakistan; 7 Dr. Saima Chaudhry, BDS, Ph.D., Adjunct Faculty, University of Health Sciences, Lahore, Pakistan. Professor Oral Pathology, Director CHPL, The University of Lahore, Lahore, Pakistan

**Keywords:** AJCC 2018, Clinical Stage, Depth of invasion, Oral Squamous Cell Carcinoma, Primary Tumor stage, Surgical margin

## Abstract

**Objective:**

To investigate the association between clinicopathological findings and tumor stage according to AJCC 2018 guidelines in patients suffering from Oral squamous cell carcinoma (OSCC).

**Methods:**

A descriptive study was conducted from January 2019 to January 2020 at King Edward Medical University and University of Health Sciences on a total of 49 patients enrolled after obtaining written informed consent. Clinical and radiographic findings were recorded. Pathological reporting was done using AJCC 2018 cancer staging guidelines. Association between clinicopathological features with tumor stage and grade was assessed using Chi-square and Kruskal-Wallis test.

**Result:**

Mean age of the patients was 46.1 ± 10.6 years. Most of the tumors were of well differentiated type (49%) and moderately differentiated (40.8%) with predominant clinical stage III in 42.9% & IV in 44.9 % and primary tumor stage pT2 28.6% & pT3 36.7%. Significant difference was seen for primary tumor stage in relation to age, gender, depth of invasion, primary site, and size of tumor (p < 0.01). For clinical stages, significant difference was observed in the age, gender, size of tumor, nodal metastasis, and anatomical tumor site (p < 0.01).

**Conclusion:**

Application of 8th Edition AJCC guidelines identifies the importance of the latest classification with strong association of latest stage criteria with age, gender, site of primary tumor, tumor thickness, depth of invasion, nodal metastasis and size of largest lymph node involved, and Level of Lymph node involved (level III & V) in a subset of patients from a developing country.

## INTRODUCTION

More than 90% oral and oropharyngeal cancers are Squamous cell carcinomas (OSCC).^1^ Despite advancement in therapies, morbidity, and mortality of OSCC has not improved significantly during last 30 years with approximately 50% of patients experiencing recurrence. Recurrent OSCC is reported to have a poor prognosis with a median survival of about 12 months.^2^ Grading and clinical staging has provided for long, a sound and reliable mode of predicting the prognosis and taking treatment decisions. Factors reported to be associated with poor prognosis of patients with OSCC are tumor stage, tumor grade, extra capsular spread, nodal metastasis, positive surgical margins, and perineural invasion.^3^

Research in the past decade, into clinicopathological features that may predict prognosis of the disease, have led to the revision of the TNM classification in American Joint Committee on Cancer (AJCC) 2018 edition to include pathological parameters of depth of invasion (DOI), extra-capsular spread, perineural, lymphovascular and worst pattern of invasion leading to reinvestigating potential of this staging system in predicting disease outcomes.^4^ In AJCC 2018, DOI of primary tumor has been incorporated into the T category and is a key component in redesigning the staging system.^5^ Primary tumor stage-I has now been upgraded to stage-II if DOI is greater than 5mm, similarly stage-II has been upgraded to stage-III with the presence of DOI greater than 10mm. Extracapsular spread (ECS) has been added to N stage.

With emergence of new classification pathologists have found it to be more elaborative and conclusive but World Health Organization (WHO) still ratifies WHO 2017 classification. Further modifications are still expected and indicative for AJCC 2018 Classification. In light of these controversies how well this new staging system can act as a predictor for factors such as prognosis, survival, occult metastasis and recurrence is not fully established and is still up for debate.

Due to scarcity of literature in the area from our region present study was conducted to analyze clinical and pathological parameters of OSCC in relation to latest staging system in the local population.

## METHODS

This cross-sectional study was conducted on 49 patients diagnosed with OSCC admitted from January 2019 to January 2020 at Oral & Maxillofacial Surgery department of King Edward Medical University, Lahore. Pathological reporting was done at the Department of Morbid Anatomy & Histopathology and Oral Pathology, University of Health Sciences, Lahore using AJCC 2018 cancer staging guidelines. Ethical approval of the study was obtained from the institutional review board (IRB) / ethical committee University of Health Sciences Lahore vide letter No. UHS/REG-18/ERG/2623 dated August 7^th^ 2018. Patients willing to participate in the study with biopsy proven OSCC irrespective of cancer stages, grades, age, or gender were included while those with history of prior radiotherapy or chemotherapy, any other malignancy or previously treated for malignancy were excluded. Patients with verrucous and basaloid OSCC were excluded based on AJCC 2018 requirement.

Patient’s age, gender, residence, history of chief complaint, sign & symptoms, risk factor, site involved, personal contact, details of biopsy proven tumor and past medical history were recorded. Seventy patients with OSCC reported within a year, out of which forty-nine fulfilled the study criteria. Initial information was obtained and recorded. For clinical staging, clinical examination was done of the head and neck region for any growth, ulcer or swelling, palpable lymph nodes and salivary glands. Intraoral inspection included lips, buccal mucosa, gingiva/alveolar ridge, maxilla, mandible, vestibule, retromolar trigone, tongue, floor of mouth, hard palate, soft palate, and oro-pharynx. Visible tumor was measured in its greatest dimension for clinical tumor and palpable lymph node for clinical nodal assessment. Examination of Chest X-rays, CT scans and MRI were done for assessing regional or distant metastasis.

Histological slides of excisional biopsy samples were independently assessed by two experienced histopathologists, neither of them were involved in the sample collection and slide preparation and were blinded to each other’s findings. Clinical and Pathological staging and grading was done using AJCC 8th Edition criteria. Cases were further sub-classified as stage I & II as Early stage and stage III & IV as Advanced stage. Variables assessed were gross tumor thickness/size, histological type and grade, depth of invasion, pattern of invasion, worst pattern of invasion, total number of regional lymph nodes involved, level of lymph nodes involved on histopathology, bone invasion, perineural invasion, lymphovascular invasion (LVI) and surgical margins.

Statistical analysis was done through SPSS statistical Package version 25.0. Nominal data was recorded as frequencies and percentages and numerical data noted as mean and standard deviation. Chi-square test was used to observe association between stages and different clinicopathological variables. Difference in scores of clinicopathological features across the primary tumor stage and clinical stages overall was assessed using the Kruskal Wallis test. *p*-value ≤ 0.05 was considered significant for all analysis keeping confidence level at 95%.

## RESULTS

Age range of OSCC patients assessed was 25-67 years, with more males as compared to females. Details of clinicopathological data are given in Table-I. The highest mean value of number of lymph nodes involved and size of the largest lymph node was in lymph node level I & II as shown in Table-II. In bivariate analysis, age, gender and tumor thickness were significantly related to both pathological tumor and clinical tumor stages. Anatomical site and nodal metastasis with Clinical stage and DOI and Primary site with Primary tumor stage were significantly associated (Table-III). The strength of association between depth of invasion (DOI) with pT was strong (epsilon square >0.14) however this difference within stages was not statistically significant. Non-significant differences were observed in the DOI scores, number of lymph nodes involved and worst pattern of invasion scores across all clinical and pathological tumor stages. The strength of association (epsilon square) between the worst pattern score of invasion and number of lymph nodes involved for both pathological/clinical stage was weak (0.069/0.028, 0.073/0.040), for DOI it was moderate (0.110/0.121), and strong (0.257) for size of tumor with pT. Mean DOI was higher for cT & pT stages III & IV respectively.

**Table-I:** Clinicopathological characteristics of patients with OSCC.

Variables (Numerical)	Mean ± SD
Age (Years)	46.16 ± 10.69
Tumor Size (cm)	5.55 ± 4.11
Depth of Invasion (mm)	8.63 ± 4.62
Worst pattern of Invasion Score	1.49 ± 1.32
Size of largest Lymph node (cm)	1.53 ± 2.66
No. of Lymph Nodes Submitted	33.57 ± 18.25
No. of Lymph Nodes Involved	2.18 ± 4.15
*Variable (Categorical)*	*n (%)*
***Gender***	
Male	34 (69.4)
Female	15 (30.6)
***cT Stage***	
cT1	2 (4.1)
cT2	18 (36.7)
cT3	19 (38.8)
cT4	10 (20.4)
***pT Stage***	
pT1	4 (8.2)
pT2	14 (28.6)
pT3	19 (38.8)
pT4	12 (24.5)
***pN***	
pN0	22 (44.9)
pN1	7 (14.3)
pN2a	2 (4.1)
pN2b	9 (18.4)
pN2c	1 (2)
pN3a	1 (2)
pN3b	4 (8.2)
pNX	3 (6.1)
***Grade***	
Poorly differentiated	5 (10.2)
Mod. Differentiated	20 (40.8)
Well differentiated	24 (49)
***Surgical margins***	
Negative	29 (59.2)
Positive	20 (40.8)
Deep	2(4.1%)
Deep + Others	8(16.3%)
Others	10(20.4%)
***Nodal Metastasis***	
No	25 (51%)
Yes	24 (49%)
***Depth of Invasion Range***	
Upto 5 mm	13 (26.5)
≥6mm-10mm	20 (40.8)
Above 10mm	16 (32.7)
***Perineural Invasion***	
No	25 (51)
Yes	24 (49)
***Lymphovascular Invasion***	
No	29 (59.2)
Yes	20 (40.8)
***Extracapsular Spread***	
No	46 (93.9)
Yes	3 (6.1)
***Clinical Stage***	
I	1 (2)
II	5 (10.2)
III	21 (42.9)
IV	22 (44.9)
***Tumor Stage***	
Early Stage (I&II)	18 (36.7)
Advance Stage (III&IV)	31(63.3)
	

**Table-II:** Total number/mean of lymph nodes submitted, involved and mean of size of the largest lymph nodes involved.

Level	No. of Lymph nodes Submitted	Lymph nodes submitted (Mean ± SD)	No. of Lymph nodes involved	Lymph nodes involved (Mean ± SD)	Size of largest lymph node (Mean ± SD)
I	363	37.39 ± 15.02	43	5.21 ± 5.37	3.08 ± 3.33
II	511	37.02 ± 15.01	30	6.00 ± 6.13	3.47 ± 3.92
III	393	37.26 ± 15.18	15	7.50 ± 7.79	2.08 ± 1.06
IV	222	35.88 ± 14.48	11	11.25 ± 7.80	1.90 ± 0.82
V	156	37.15± 13.90	08	11.00 ± 10.59	1.80 ± 0.98

**Table-III:** Clinical stage overall and primary tumor stages across significant numerical clinicopathological variables.

Clinicopathological variables	Primary Tumor Stage (Pt)	N	Mean rank	P value		Epsilon square η2	Clinical stage		N		Mean Rank	P value	Epsilon square η2
Age	pT 1	4	23.25	0.039		0.142	Stage I		1		47.5	0.024	0.199
	pT 2	14	32.11				Stage II		5		30.10		
	pT 3	19	18.24				Stage III		21		29.36		
	pT 4	12	28.00				Stage IV		22		18.66		
Tumor thickness	pT 1	4	13.13	0.002		0.257	Stage I		1		15.50	0.045	0.113
	pT 2	14	15.50				Stage II		5		8.60		
	pT 3	19	28.76				Stage III		21		27.33		
	pT 4	12	34.08				Stage IV		22		26.93		
Worst pattern of invasion score	pT 1	4	23.50	0.323		0.069	Stage I		1		39.50	0.343	0.073
	pT 2	14	20.46				Stage II		5		26.70		
	pT 3	19	25.37				Stage III		21		21.50		
	pT 4	12	30.21				Stage IV		22		27.30		

*Clinicopathological Parameters*			*Primary Tumor stage (pT) n (%)*									*P value*	

			*pT 1*		*pT 2*			*pT 3*		*pT 4*			

Gender	Male		2 (50%)		5 (35.7%)			17 (89.5%)		10 (83.3%)		0.007	
	Female		2 (50%)		9 (64.3%)			2 (10.5%)		2(16.7%)			
Depth of invasion	Upto 5mm		3 (75%)		4 (28.6%)			4 (21.1%)		2 (16.7%)		0.021	
	>5mm-10mm		0 (0%)		7 (50%)			11 (57.9%)		2 (16.7%)			
	Above 10mm		1 (25%)		3 (21.4%)			4 (21.1%)		8 (66.7%)			
Primary site	Lip		0 (0%)		0 (0%)			0 (0%)		1 (8.3%)		0.002	
	Alveolar ridge		0 (0%)		1 (7.1%)			1 (5.3%)		6 (50%)			
	Buccal mucosa		2 (50%)		4 (28.6%)			12 (63.2%)		2 (16.7%)			
	Hard palate		0 (0%)		0 (0%)			0 (0%)		2 (16.7%)			
	Tongue		2 (50%)		9 (64.3%)			6 (31.6%)		1 (8.3%)			

*Clinicopathological Parameters*			*Clinical stage n (%)*									*P value*	

			*I*		*II*			*III*		*IV*			

Gender	Male		0 (0%)		2 (5.9%)			12(35.3%)		20 (58.8%)		0.006	
	Female		1 (6.7%)		3 (20%)			9 (60%)		2 (13%)			
Nodal metastasis	Negative		1 (4%)		5 (20%)			12 (48%)		7 (28%)		0.025	
	Positive		0 (0%)		0 (0%)			9 (37.5%)		15 (62.5%)			
Anatomic Site	Anterior		0 (0%)		0 (0%)			1 (100%)		0 (0%)		0.001	
	Middle		0 (0%)		5 (11.4%)			17(38.6%)		22 (50%)			
	Posterior		1 (50%)		0 (0%)			1 (50%)		0 (0%)			
	Anterior and middle		0 (0%)		0 (0%)			2 (100%)		0 (0%)			

pN was observed to be associated with positive surgical margins, primary tumor site, lymph node level, and ECS. LVI was independently related with positive surgical margins. Difference of means for the age, size of tumor, level of lymph nodes involved (Level III & V), and size of the largest lymph nodes involved across early and advance stage tumors was also statistically significant.

Difference in means is seen across the different surgical margin types for Worst Pattern of Invasion scores (p=0.049) whereby significant difference in means were seen between positive margins in comparison to no margins involvement with p < 0.001 using Kruskal Wallis test.

## DISCUSSION

Current study was carried out to investigate the association of clinicopathological features of OSCC with clinical and pathological tumor stage according to AJCC 2018 criteria. In the present study OSCC is more common in males than females (2.2:1) which aligns with the existing data about the predisposing factors usage. There is excessive use of cigarettes, smokeless tobacco, and alcohol in males as compared to females. According to global adult tobacco survey younger males smoke fifty times more than females. Use of smokeless tobacco alone is reported in a GATS survey of Pakistan to be more prevalent in males.^6^ This data is in accordance with other Pakistani and African studies.^7^ Age also plays a vital role in determining the overall prognosis and survival of the disease. AJCC staging system has linked age with early and late stages of OSCC previously,^8^ same is also reported in our study. Literature also reports possible genetic predilection and reason for early age onset of OSCC hence a need for possible genetic polymorphism evaluation in populations.^9^ In the current study the most common site for the primary tumor is seen to be buccal mucosa which is in accordance with the literature.^10^ Santos et al (2016) mentioned association of anatomical site with advanced clinical stage which is similar to our study with more cases involving intraoral sites than the lip.^11^ Many studies have provided evidence of link between anatomical sites and survival in OSCC patients.^12^,^13^

Tumor thickness was significantly related with both clinical and pT Stage in present study with p 0.04 and 0.002 respectively. Literature reports importance of this variable but in conjunction with other variables as part of multivariate analysis for predicting the survival rate of these patients.^14^ Some studies have reported tumor thickness as an independent risk factor for determining the nodal metastasis.^15^ Similar to previous literature no association was observed between grades and other clinicopathological variables. This may be attributed to subjective nature of evaluation and small size of biopsies with high heterogeneity. TNM staging in OSCC can provide us with significant information about cervical lymph node metastasis which is an important predictor of overall survival.^16^

Nodal metastasis is seen in 52.2% of our OSCC cases which is close to a study by Arain A et al (2020) where equal number of cases are under analysis with similar grading in tumors.^17^ The risk of nodal metastasis is reported to be lower in T1 and T2 tumors but much higher in T3 and T4^17^ similar to our study which signifies less likelihood of having nodal metastasis at early stages in comparison to late stages. However, contradictory findings have also been reported in literature with occurrence of nodal metastasis in early-stage tumors with 30% of the N0 tumors having occult metastasis.^17^ This emphasizes that clinically and radiographically proven negative nodes cannot be exempted and need biopsy before deciding to rule out neck dissection as a treatment option in patients with OSCC.

In the present study there was a statistically significant difference in the number of lymph nodes involved across the pathological tumor stage which is comparable to another local study thus confirming the findings.^18^ Alsaffar et al in their study on assessment of depth of invasion in tongue OSCC reported the pathological depth of invasion to be greater than the clinical depth.^19^ These results were comparable to our study where difference in depth of invasion across the clinical and Primary Tumor stage was clear supporting the clinical decision making based on pathological rather than clinical data.^20^ Locoregional metastasis in relation to tumor thickness shows metastatic rate of 6.5% in lesions measuring less than 2cm while those larger than 2cm showed a metastatic rate of 30%.^21^ However, association between tumor thickness and primary tumor staging has not been studied in the past. We found greatest size of the lesion in pT III followed by stage IV while the least was seen in stage II patients.

The worst pattern of invasion (WPOI) is cited to be of a good prognostic value. Highest percentage of disease progression on follow-up has been seen in patients with the WPOI 4 followed by WPOI-5 while the least in patients with WPOI 1.^22^ Current study evaluated the association between worst pattern of invasion score and pathological and clinical tumor staging. The highest WPOI score was seen in Primary Tumor stage IV followed by stage III and the least in Stage-I. This identifies WPOI 4 & 5 as possible predictors of poor survival where they reflect advance stage disease also in cohesion with Mishra A et al where WPOI is identified as independent prognostic factor for OSCC.^23^ They also reported association of WPOI 4 with surgical margins, poor disease free survival and overall survival however our study did not include survival analysis but is in cohesion in terms of surgical marginas involvement thereupon surgeons may involve larger healthy margin especially in cases with initial histological assessment reporting Invasive pattern in order to avoid loco-regional recurrence and improve survival.^23^

In this study, maximum involved margins were seen in tongue, buccal mucosa, and alveolar ridge, with 59% of the patients having clear margins similar to study by Sutton et al. with 53% clear margins.^24^ Previous studies have reported floor of mouth cancers with higher frequency of involved margins then tongue OSCC^25^,^26^ These differences can be attributed to the individual patient and surgeon variations along with difference in sample sizes.

There is scarcity of regional data assessing relationship between newly incorporated clinicopathological factors with the latest AJCC staging system for accurate staging and grading of OSCC tumors and also determining these factors as reliable histopathological parameters. Age, gender, tumor thickness, primary site/ anatomical sites are important variables of Clinical & Primary tumor staging systems. DOI shows strong association with pT reflecting its usefulness in better categorization and grading of OSCC tumors which may lead to accurate diagnosis and treatment strategies. WPOI 4 & 5 strong association with late pT stages and positive surgical margins recommends agressive treatment to prevent relapse and improve survival.

### Limitations

Limitations of the study are a comparatively smaller sample size, single centre study and limited post-operative information due to loss to follow up caused by unavailability of patients either due to death or inaccessibility as patients came from distant places and are mostly from poor socio-economic background. This makes it difficult for continuity of care in a specific center, so data obtained in setting of developing countries like Pakistan is generally sporadic as was the case in our study.

## CONCLUSION

Present study validates the importance of primary tumor stage in assessing the prognosis of OSCC. Primary tumor stages, clinical stage, early & advance stages, and pathological nodal stage are all strongly linked to histopathological parameters in OSCC. Using AJCC 2018 cancer staging system strong associations were found between clinical staging and primary tumor with the parameters of age, gender, size of tumor, primary site of tumor, nodal metastasis, depth of invasion, level of lymph nodes and size of the largest lymph nodes involved. Future research may incorporate longitudinal data sets and prognostic parameters to further validate the current findings.

### Authors Contribution:

**NRK & SC:** Concept of work, acquisition & analysis of data, drafting manuscript, critical revision, final approval and Agreement to be accountable for all aspects of the work.

**NN & SK:** Histopathological analysis, critical revision and agreement to be accountable for all aspects of the work.

**NR & AI:** Acquisition of data, manuscript editing and critical revision.

**RA:** Analysis of data and critical revision.
